# Contribution of Ultra Deep Sequencing in the Clinical Diagnosis of a New Fungal Pathogen Species: *Basidiobolus meristosporus*

**DOI:** 10.3389/fmicb.2017.00334

**Published:** 2017-03-07

**Authors:** Emilie Sitterlé, Christophe Rodriguez, Roman Mounier, Julien Calderaro, Françoise Foulet, Michel Develoux, Jean-Michel Pawlotsky, Françoise Botterel

**Affiliations:** ^1^Département de Microbiologie, Créteil, Dynamyc, ENVA, UPECCreteil, France; ^2^Département de Microbiologie, Next-Generation Sequencing Platform pACT, IMRB CréteilFrance; ^3^Institut Mondor de Recherche Biomédicale U955Créteil, France; ^4^Réanimation ChirurgicaleCréteil, France; ^5^Département de PathologieCréteil, France; ^6^Département de Parasitologie - MycologieParis, France

**Keywords:** Ultra-Deep Sequencing, fungal infection, gastro-intestinal basidiobolomycosis, *Basidiobolus meristosporus*, ITS amplicons

## Abstract

Some cases of fungal infection remained undiagnosed, especially when the pathogens are uncommon, require specific conditions for *in vitro* growth, or when several microbial species are present in the specimen. Ultra-Deep Sequencing (UDS) could be considered as a precise tool in the identification of involved pathogens in order to upgrade patient treatment. In this study, we report the implementation of UDS technology in medical laboratory during the follow-up of an atypical fungal infection case. Thanks to UDS technology, we document the first case of gastro-intestinal basidiobolomycosis (GIB) due to *Basidiobolus meristosporus*. The diagnosis was suspected after histopathological examination but conventional microbiological methods failed to supply proof. The final diagnosis was made by means of an original approach based on UDS. DNA was extracted from the embedded colon biopsy obtained after hemicolectomy, and a fragment encompassing the internal transcribed spacer (ITS) rDNA region was PCR-amplified. An Amplicon library was then prepared using Genome Sequencer Junior Titanium Kits (Roche/454 Life Sciences) and the library was pyrosequenced on a GS Junior (Roche/454 Life Sciences). Using this method, 2,247 sequences with more than 100 bases were generated and used for UDS analysis. *B. meristosporus* represented 80% of the sequences, with an average homology of 98.8%. A phylogenetic tree with *Basidiobolus* reference sequences confirmed the presence of *B. meristosporus* (bootstrap value of 99%).

**Conclusion** : UDS-based diagnostic approaches are ready to integrate conventional diagnostic testing to improve documentation of infectious disease and the therapeutic management of patients.

## Introduction

Fungal infections can be challenging when the pathogen is uncommon, requires specific *in vitro* growth conditions, or is part of a multimicrobial community. Therefore, some cases of fungal or other infectious diseases remain undiagnosed despite extensive clinical laboratory investigations. In such conditions, Ultra Deep Sequencing (UDS) appears to be a good compromise to improve laboratory identification of involved pathogens and subsequently adapt their therapeutic treatment (Wilson et al., 2014).

Our aim was to show how diagnosis of infection in a polymicrobial sample could be achieved when conventional microbiological methods fail. We report the implementation of UDS technology in medical laboratory work during the follow-up of an unusual fungal infection case and with which the first diagnosis of gastrointestinal basidiobolomycosis (GIB) caused by *Basidiobolus meristosporus* was reached.

## History

**Figure 1:**
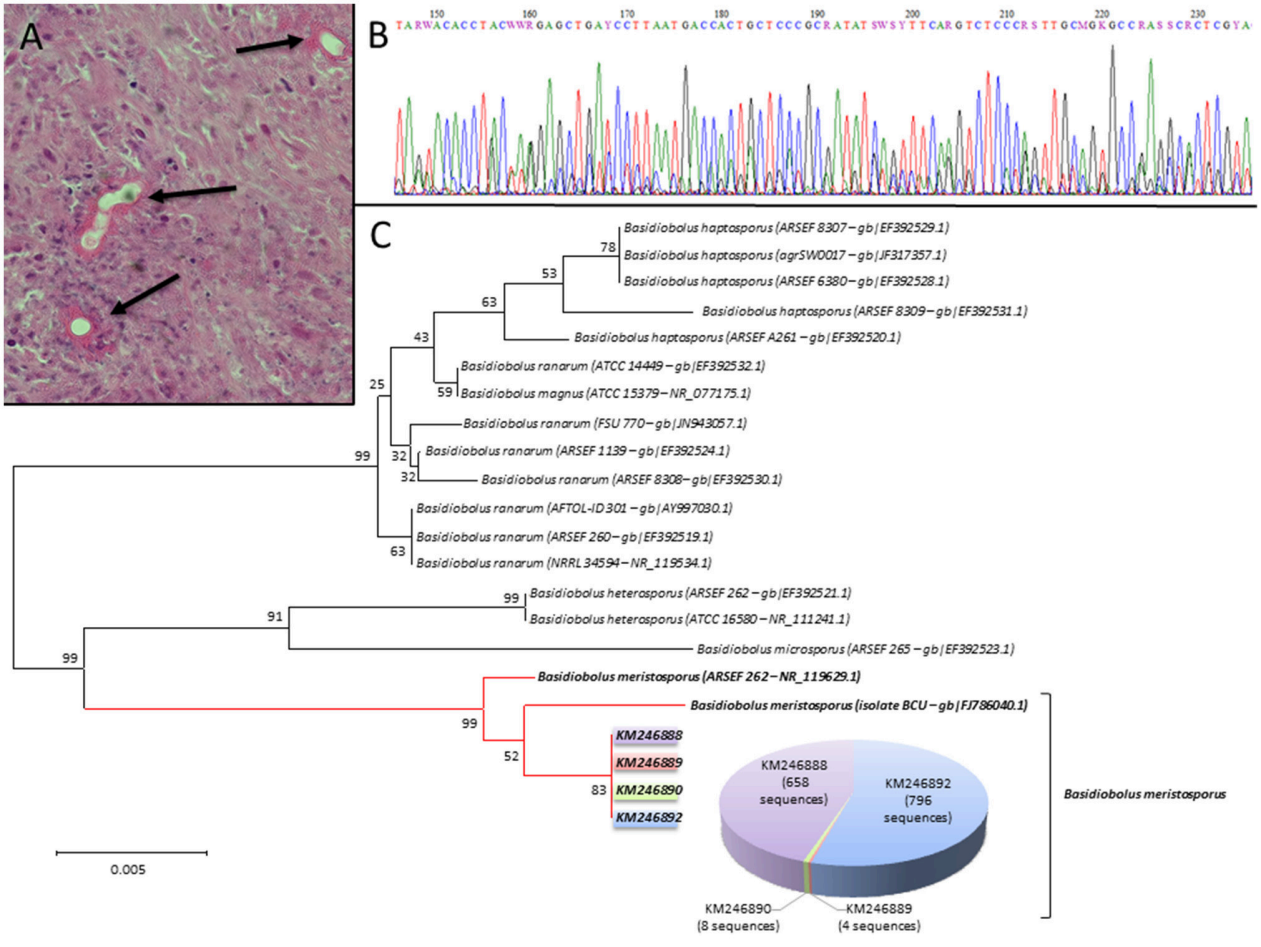
**(A)** Colon biopsy stained with Periodic acid-Schiff stain showing several hyphal structures (black arrows) surrounded by eosinophilic-rich infiltrate called Splendore-Hoeppli phenomenon (magnification × 400). **(B)** ITS2 Sanger electropherogram showing superposed sequences, suggesting the presence of different fungi in the sample. **(C)** A Neighbor-Joining tree generated for ITS2 rDNA region sequences identified as *Basidiobolus meristosporus* (accession number KM24688 to KM246892) with *Basidiobolus* spp. sequences references using a CLUSTAL X alignment with the optimal criteria set for Distance in MEGA6. Percentage at nodes indicates bootstrap values for 1,000 replicates. Number of sequences that represented our four sequences is indicated in the pie chart (only sequences representing at least three sequences among *Basidiobolus* bunch of sequences are indicated).

A 41-year-old female, native and living in Cameroon without any past history of travel to other countries, was presented with abdominal pain, which started 2 months prior to her hospital admission. The patient was HIV-positive (330 CD4/mm^3^), on HAART treatment, and her disease was well controlled (undetectable HIV RNA load). An abdominal CT-scan revealed a large mass in the right lower abdomen, which involved the ascending colon, the terminal ileum and the bladder. An emergency laparotomy showed that the mass was highly inflammatory and had pseudotumoral appearance with a fistula. On that occasion, the patient underwent a right hemicolectomy, Hartmann procedure, bladder suture, and Miculicz drainage. Histopathological examination and conventional microbial cultures of the surgical specimen were performed. The main results of the histopathological examination, upon using Periodic acid-Schiff staining, showed large hyphae surrounded by a thick eosinophilic material (Splendore-Hoeppli phenomenon) (Figure 1A). A presumptive diagnosis of fungal infection possibly by *Basidiobolus* sp. was made though no positive culture of this fungus was obtained. *Basidiobolus* spp. cannot be isolated if sample is stored at 4°C or if Sabouraud agar media contain antibiotics (Al-Naemi et al., 2015; Geramizadeh et al., 2015; Shaikh et al., 2016). Futhermore, hyphae of *Basidiobolus* spp. may be easily damaged during tissue homogenization processing. The patient received post-op liposomal amphotericin B (5 mg/kg/day) and fluconazole (400 mg/day) for 10 days followed by oral and IV itraconazole (200 mg × 2/day), but she died 3 months later after several episodes of septic shock.

## Methodology of the diagnosis

This study was carried out in compliance with the Helsinki declaration. A written consent form was obtained from the family for publication.

After the surgery, a DNA extraction from a formalin-fixed, paraffin-embedded tissue biopsy, obtained from the excised colon, was made using a BioRobot EZ1 (Qiagen), and followed by amplification and Sanger sequencing of the internal transcribed spacer (ITS) regions of the fungal rDNA (White et al., 1990). The results of ITS sequencing remained inconclusive because of superposition of several electropherograms, suggesting the coexistence of several fungal species (Figure 1B). Thus, we decided to use UDS to identify the culprit fungal pathogen within this polymicrobial sample, and for such analysis the ITS2 region was PCR-amplified. An Amplicon library was then prepared using GS Junior Titanium Kits (Roche/454 Life Sciences) and the library was pyrosequenced on a Genome Sequencer Junior (Roche/454 Life Sciences). The generated data were analyzed with PyroMIC (protected software, IDDN FR.001.400018.000.S.P.2014.000.31230) which contains a cleaned fungal database created from ITS NCBI fungal sequences. Ultimately, only sequences of >100 bp, with ≥98% homology, and 0.0 *e*-value were considered for species identification. Haplotypes of sequences of interest were done by means of CD-HIT software (Li and Godzik, 2006; Ghannoum et al., 2010; Findley et al., 2013). A nucleotide alignment, by CLUSTAL X, was used to build a dendrogram using the Neighbor Joining, Kimura 2 parameter method, 1,000 bootstrap, with the MEGA6 software (Tamura et al., 2013).

As for the UDS method, 2,247 sequences (matching quality criteria) were used for analysis. For identification at the species level, the software considered 1,832 sequences with an average length of 440 bp. Overall, four different species were identified, of which *B. meristosporus* represented 80% (1,466/1,832) of the sequences, *Malassezia globosa* 7.2% (1,32/1,832), *Malassezia restricta* 6.8% (124/1,832), and *Candida zeylanoides* 6% (110/1,832), with an average homology of 98.8, 98.7, 99.0, and 99.0% respectively. Among the *B. meristosporus* sequences, four haplotypes were found and considered as representative sequences (NCBI Accession number: KM246888, KM246889, KM246890, KM246892) to construct a Neighbor Joining tree with all *Basidiobolus* spp. references that are available on NCBI. This tree showed a clusterization of our sequences with the two reference sequences of *B. meristosporus* (ATCC 14450 and isolate BCU1), and the bootstrap value was 99% (Figure 1C). Eventually, this approach enabled us to identify *B. meristosporus* as the causative pathogen of the GIB in this patient.

## Discussion

We describe here the first human diagnosis of GIB due to *B. meristosporus* and the capacity of UDS-based diagnostic approach to detect fungal pathogen in a polymicrobial sample. As a result of the increase of immunocompromised patients and the emergence of new fungal pathogens, laboratories are facing diagnostic challenges to identify rare pathogens or pathogens implicated in polymicrobial samples (Irinyi et al., 2015). Basically, when a presumptive diagnosis of an infection is provoked, early detection and identification of the etiologic agent permits the initiation of appropriate antifungal therapy to reduce morbidity. When culture is possible, the fungal identification is made by morphological and biochemical methods, which are time-consuming and require trained lab personnel. Alternatively, molecular methods such as DNA barcoding, on isolated colonies, offer a practical approach for species identification (Irinyi et al., 2015). In culture negative cases, sequencing of a PCR product from tissue could be performed as an alternative means of diagnosis (El-Shabrawi et al., 2011). However, the presence of several coexisting species may hamper sequence reading.

In this context, the UDS has been integrated in the diagnostic process to identify such unusual fungal within the polymicrobial sample. GIB is a very rare emergent fungal infection caused by *Basidiobolus* spp. Although several species of *Basidiobolus* have been described in the literature (Gryganskyi et al., 2012), *Basidiobolus ranarum* was the only one reported in human gastrointestinal cases (Lyon et al., 2001). Nevertheless, in the few published cases, the diagnosis was based on histopathology examination and rarely on tissue culture or serodiagnosis. Indeed, in the review of the 44 cases of GIB published by Vikram et al., *Basidiobolus* spp. was isolated in culture from only of 24 patients for which a sample was available. In this review, the specific serum antibodies to *B. ranarum* were detected in only 8 (50%) of the 16 patients who underwent testing (Vikram et al., 2012). Currently molecular identification is the only method that gives a better species identification and since 2012, the *Basidiobolus* clade has included six distinct species which are closely related to each other (Gryganskyi et al., 2012). Accordingly, we can suppose that some reports on infections documented as *B. ranarum*-induced might be due to other species.

In the presented case, the histopathology result suggested *Basidiobolus* sp. but conventional diagnostics by culture or molecular approaches failed to identify the *B. meristosporus*, which was detected thanks to UDS. Only the detection of fungi by UDS approach would have provided conclusive information to give an adapted treatment. Furthermore, the sensitivity of the UDS methodology enabled the identification of additional yeast species (*C. zeylanoides, M. globosa*, and *M. restricta*), which were considered as either potentially implicated in the pathology or as sample contaminants (skin or gut biota) (Gouba et al., 2013). In brief, despite the availability of several conventional microbiological diagnostics, it will be interesting to use UDS with targeted approach for the polymicrobial samples diagnosis in some particular cases: (i) when the clinical presentation is atypical or when the patient is immunocompromised or comes from tropical countries, (ii) when the culture remain sterile despite positive histopathology or positive direct examination; (iii) for pathogens uncultivable or after the beginning of treatment (iv) to confirm the presence of pathogens requiring specific treatment or when the treatment incurs serious secondary effects (benefit-risk).

Moreover, free software versions are available to help interpret metagenomics data and the cost could be drastically decreased in the future with the development of UDS sharing platform. We believe that this approach is ready to integrate conventional diagnostic investigations to improve documentation of infectious disease and the therapeutic management of patients.

## Author contributions

Conceptualization: FB, ES, and CR. Methodology: FB, ES, CR, and JMP. Software: ES and CR. Validation: FB and ES. Formal analysis: FB, ES, and CR. Investigation: FB. Resources: RM, JC, FF, and MD. Writing (original draft preparation): FB, ES, CR, and JMP. Supervision: FB.

### Conflict of interest statement

The authors declare that the research was conducted in the absence of any commercial or financial relationships that could be construed as a potential conflict of interest.
